# Maternal Separation Combined With Limited Bedding Increases Anxiety-Like Behavior and Alters Hypothalamic-Pituitary-Adrenal Axis Function of Male BALB/cJ Mice

**DOI:** 10.3389/fnbeh.2020.600766

**Published:** 2020-11-12

**Authors:** Rodrigo Orso, Kerstin Camile Creutzberg, Erika Kestering-Ferreira, Luis Eduardo Wearick-Silva, Saulo Gantes Tractenberg, Rodrigo Grassi-Oliveira

**Affiliations:** ^1^Developmental Cognitive Neuroscience Lab (DCNL), Pontifical Catholic University of Rio Grande do Sul (PUCRS), Porto Alegre, Brazil; ^2^Brain Institute (InsCer), Pontifical Catholic University of Rio Grande do Sul (PUCRS), Porto Alegre, Brazil; ^3^Department of Pharmacological and Biomolecular Sciences, University of Milan, Milan, Italy

**Keywords:** maternal separation, limited bedding, HPA axis, anxiety-like behavior, CRH, corticosterone

## Abstract

Early life stress (ELS) is considered a risk factor for the development of psychiatric conditions, including depression and anxiety disorder. Individuals that live in adverse environments are usually exposed to multiple stressors simultaneously, such as maternal neglect, maltreatment, and limited resources. Nevertheless, most pre-clinical ELS models are designed to explore the impact of these events separately. For this reason, this study aims to investigate the effects of a combined model of ELS on anxiety-like behavior and hypothalamic-pituitary-adrenal (HPA) axis related targets. From PND 2 to PND 15 BALB/cJ mice were exposed simultaneously to maternal separation (MS; 3 h per day) and limited bedding (LB; ELS group) or left undisturbed (CT group). Maternal behavior was recorded in intercalated days, from PND 1 to PND 9. Male offspring were tested for anxiety-like behavior from PND 53 to PND 55 in the open field test (OF), elevated plus-maze (EPM), and light/dark test (LD). After behavioral testing, animals were euthanized, and glucocorticoid receptor (*Nr3c1)*, corticotrophin-releasing hormone (*Crh*), and its receptor type 1 (*Crhr1*) gene expression in the hypothalamus were measured. Moreover, plasma corticosterone levels were analyzed. We observed that ELS dams presented altered quality of maternal care, characterized by a decrease in arched-back nursing, and an increase in passive nursing. Stressed dams also showed an increase in the number of exits from the nest when compared to CT dams. Furthermore, ELS animals showed increased anxiety-like behavior in the OF, EPM, and LD. Regarding gene expression, we identified an increase in hypothalamus *Crh* levels of ELS group when compared to CT animals, while no differences in *Nr3c1* and *Crhr1* expression were observed. Finally, stressed animals showed decreased levels of plasma corticosterone when compared to the CT group. In conclusion, we observed an alteration in maternal behavior in ELS dams. Later in life, animals exposed to the combined model of ELS showed increased levels of anxiety-like behavior. Moreover, the central and peripheral HPA measures observed could indicate a dysregulation in HPA function provoked by ELS exposure.

## Introduction

Over the years, animal studies have been trying to replicate human early life adverse conditions to model the impact of chronic stress exposure during sensitive periods of development. The use of preclinical research is an essential tool to uncover the neurobiological underpinnings associated with early life stress (ELS) exposure. Despite several decades of animal research, ELS studies continue to face challenges, especially because there are controversial and underpowered findings (Tractenberg et al., 2016; Bonapersona et al., 2019; White and Kaffman, 2019; Wang et al., 2020). For example, the meta-analysis of Wang et al. (2020), reported that mice exposed to maternal separation (MS) showed no alteration in anxiety-like behavior, highlighting that MS studies face some uncertainties regarding publication bias (e.g., methodological and experimental procedures inconsistencies) that might affect replication and translational potential of studies. These inconsistencies in experimental ELS protocols may induce distinct degrees of stress exposure, attenuating or exacerbating the effects on both offspring and dams (Lehmann and Feldon, 2000; Tractenberg et al., 2016).

Classical ELS models were designed to separately explore the characteristics of chronic stress early in life. For example, MS is projected to trigger fear in the offspring and dam, mainly due to exposure to an unfamiliar environment (White and Kaffman, 2019). Nevertheless, a past study has shown that maternal care increases post-MS, which could attenuate the long-term effects of MS on behavioral and biological outcomes (Orso et al., 2019). On the other hand, limited bedding (LB) generates high levels of deprivation from acceptable maternal care and resources, which are both necessary for offspring thermoregulation and adequate development (Rice et al., 2008; McLaughlin et al., 2014). We believe that the combination of these protocols is not simple to add stressors, but they can have complementary roles that could increase the challenging conditions imposed to both the dam and the offspring. For example, the pattern of maternal care post-MS reunion could be fragmented by the necessity of the dam to locate resources during LB conditions, which increases the frequency of exits from the nest (Walker et al., 2017). Furthermore, humans that live in stressful environments are usually exposed to multiple stressors simultaneously, and not one negative stimulus exclusively (Evans and English, 2002; Evans and Kim, 2007). Recently, Peña et al. (2017) reported that mice simultaneously exposed to MS and LB showed enhanced susceptibility for the development of depressive-like behavior later in life. However, how a translational approach using the combination of classical ELS models can affect stress-related parameters, such as anxiety-like behavior and hypothalamic-pituitary-adrenal (HPA) axis functioning is yet to be characterized.

Under physiological conditions, the HPA axis is activated to restore cell homeostasis following an acutely stressful event (Kapoor et al., 2008). Briefly, the synthesis and release of corticotrophin-releasing hormone (CRH) in the paraventricular nucleus of the hypothalamus will stimulate the release of adrenocorticotrophic hormone from the anterior pituitary gland, which causes the adrenal cortex to elevate glucocorticoid levels (cortisol in humans and corticosterone in rodents; Anacker et al., 2014; Jiang et al., 2019). This alteration in glucocorticoid concentration and activation of its receptors (glucocorticoid and mineralocorticoid) triggers a negative feedback mechanism, which reduces the activity of the HPA axis following the stressful event (Herman and Cullinan, 1997; Raubenheimer et al., 2006). These processes are considered essential for the regulation of stress in a healthy organism, but chronic stress may lead to dysfunctions in this stress response mechanism (Dieleman et al., 2015).

Previous studies have shown that CRH and its type 1 high-affinity receptor (CRHR1) play a major role in the pathophysiology of anxiety-like behavior (Müller et al., 2003; Wang et al., 2012). Alterations in CRH levels following ELS exposure have also been associated with long-term cognitive impairments (Ivy et al., 2010). Also, the quality of maternal care, which is usually altered during periods of ELS, appears to have a direct impact on CRH expression (Korosi and Baram, 2009). It has been previously shown that animals reared by dams with increased levels of active arched-back nursing showed reduced CRH levels in the hypothalamus and increased GR expression in the hippocampus when compared to dams with a low frequency of arched-back nursing (Plotsky et al., 1993; Francis et al., 1999; Meaney, 2001).

Despite a growing body of evidence reporting the impact of ELS on anxiety-like behavior and its relation with CRH expression, there are still challenges to improve the translational impact of ELS animal models. Adverse rearing environments in humans are usually characterized by a combination of multiple stressors simultaneously, such as abuse, neglect, household chaos, and lack of resources. However, classical ELS models that have been proposed are insufficient to cover all these conditions. For this reason, we believe that our study will help the field of ELS to move forward by contributing to the validity of the novel ELS model. To do this, we sought to investigate: (i) the effects of MS and LB combined on maternal behavior; (ii) anxiety-like behavior during young adulthood in three tasks (OF, EPM and LD); and (iii) *Crh*, *Crhr1*, and *Nr3c1* mRNA expression in the hypothalamus, as well as peripheral corticosterone levels.

## Materials and Methods

### Animals

**Figure 1:**
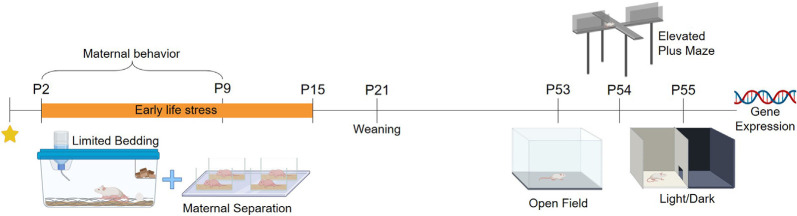
Experimental design and study outcomes.

BALB/cJ mice were acquired from the colony of the Center for Experimental Biological Models (CeMBE) at the Pontifical Catholic University of Rio Grande do Sul, Brazil (PUCRS). Animals were kept in the animal facility of PUCRS, under controlled temperature 21 ± 1°C in ventilated Plexiglas cages in a 12 h/12 h light-dark cycle (lights on at 7 AM to 7 PM). Animals had access to food and water *ad libitum* during the whole experiment. Mice were bred in-house to avoid any stress associated with animal transportation. Male BALB/cJ mice were housed with two females BALB/cJ for 72. Following this period males were removed, and females were left in pairs for 15 days. Single-housed females were checked twice every day for the presence of pups, and when a pup was found it was considered postnatal day (PND) 0.14 litters were used in total, seven litters were randomly assigned to the control group (CT), and another sevenpotential litter effects a maximum of to the stressed group (ELS). Litters were weaned at PND 21 and weighed at PND 2, PND 16, and PND 53. After weaning, three males from the same rearing protocol were grouped per cage. To avoid potential litter effects a maximum of two males per litter were used in this study, while the exceeded males and all the females were redirected to ongoing projects in the laboratory. Cage cleaning was performed once per week. Mice were tested from PND 53 to PND 55 and were euthanized 30 min after the last behavior test for brain and blood collection. The experimental design is summarized in Figure 1 (Created with BioRender.com). This study was approved by the local Committee of Ethics on the Use of Animals (CEUA) under #8546 and conducted following the Guide for the Care and Use of Laboratory Animals from the National Institute of Health (NIH).

### Early Life Stress Protocol

Animals from the ELS group were exposed to a combined stress protocol from PND2 to PND15 (Peña et al., 2019). The protocol consisted of continuous LB (Rice et al., 2008), where stressed animals were placed in cages with an aluminum mesh on the top of a small number of wood shavings, and were given only 1 g of cotton for nest building. Wood shavings were used for the pups not to get trapped under the aluminum mesh. Animals from the CT group were placed in standard cages with a regular amount of wood shavings and 4 g of cotton for nesting material. After PND15, ELS animals were returned to CT rearing conditions. On top of that, offspring was simultaneously separated from the dams for 3 h per day (9 AM to 12 PM). The dams were removed from the home cage and placed in a new cage, while the pups were individually separated in small containers with little amounts of wood shavings. During separation, the animals were kept in a separate room to avoid possible ultrasonic dam-pup vocalization. Pups were placed on a heating pad with controlled temperature (32 ± 3°C) to prevent stress-induced by hypothermia (Tractenberg et al., 2016). After the MS period, animals were returned to the home cage. Animals from the CT group were left undisturbed during the stress protocol.

### Maternal Behavior Observations

Maternal behavior observations were conducted before MS (8 AM to 9 AM), 30 min after MS (1 PM to 2 PM), and 300 min after MS (5 PM to 6 PM). Maternal behavior of all seven litters per group was recorded in intercalated days from PND1 to PND9 (PND1, 3, 5, 7, and 9). The observations were made every 2 min, which consists of a total of 90 observations per day. The following maternal behaviors were recorded: arched-back nursing, passive nursing, licking/grooming, and nest building. The following non-maternal behaviors were recorded: eating, drinking, and self-grooming. Moreover, the number of exits from the nest was recorded during the entire observation period. Two independent observers separately scored maternal behavior at different time points, which were later combined for data analysis. Total maternal and total non-maternal behavior data were calculated using the sum of all individual maternal and non-maternal behaviors recorded. Behaviors are presented by day and time of maternal behavior recording or as the sum of each behavior throughout the blocks and days.

### Anxiety-Like Behavior

Open field (OF), elevated plus maze (EPM), and light-dark (LD) were performed between 9 AM to 12 AM to evaluate the anxiety-like behavior of male offspring. To avoid potential confounding effects related to task reactiveness, we performed the behavioral battery in the same order for all animals. Moreover, ten animals per group were subjected to behavioral testing on PND53, PND54, and PND55 respectively. The three tests were video-recorded and later analyzed using the ANY-maze software (Stoelting, Company, Chicago, IL, USA). In every test, the apparatus was cleaned between animals with ethanol 70%.

#### Open Field Test

Mice were allowed to freely explore a clear acrylic arena (30 × 30 cm) for 10 min under a light intensity of 150 lux. Animals were placed in one corner of the arena to start the test. Using the above-mentioned software, the arena was divided into 16 equal squares, the center four squares were considered the center zone and the remaining squares were considered the periphery zone (Seibenhener and Wooten, 2015). The total distance traveled (in meters), the time spent in each zone, and the number of entries in each zone were analyzed. Decreased time in the center zone and fewer entries in the center zone are associated with increased anxiety-like behavior. Distance traveled was used as a measure of locomotor activity.

#### Elevated Plus Maze

For the EPM test, animals were allowed to explore a black acrylic apparatus for 5 min (Komada et al., 2008). The apparatus was 50 cm elevated from the ground and consisted of two open arms (30 × 5 cm each) and two closed arms (30 × 5 cm, with a 15 cm wall, each). The test was performed under a light intensity of 40 lux. Mice were placed in the center of the maze heading one of the open arms. The time spent in each arm, the number of entries in each arm, and the number of head dips in the open arms was evaluated. Decreased time in the open arms and fewer entries in the open arms are associated with increased anxiety-like behavior. Moreover, a head dip in the open arm to investigate under the maze is considered a risk assessment behavior.

#### Light/Dark Test

The LD apparatus consisted of two acrylic chambers (21 × 21 × 20 cm), one clear chamber and one black chamber, connected by an open door (Bourin and Hascoët, 2003). The clear chamber was bright (390 lux), while the black chamber was dark. Animals were placed in the bright chamber and had 10 min to freely explore the whole apparatus. The time spent in each chamber, the number of transitions between chambers, and the number of head pokes between the dark and bright chamber were analyzed. Decreased time spent in the light zone and the number of transitions is associated with increased anxiety-like behavior. Furthermore, a head poke occurs when the animal explores a different chamber of the apparatus through the connecting door, which is considered a risk assessment behavior.

### *Z*-Score

The *z*-score combines data from all behavioral tests. The following behavior measures were used: time spent in the center of the OF; time spent in the open arms of the EPM; and time spent in the bright chamber of the LD. In each test the individual *z*-score was calculated using the formula: (y — x)/z, where *y* is the animal score, × is the average, and *z* is the standard deviation. Finally, the average of the three *z*-scores was calculated and analyzed. A higher *z*-score represents an increase in anxiety-like behavior.

### Gene Expression Analysis

Animals were euthanized by decapitation 30 min after the LD test and the whole brain was removed. The hypothalamus was immediately hand-dissected and snap-freeze in dry ice. The samples were then stored in −80°C for further molecular analysis. Total RNA was extracted from 8 to 10 samples per group using QIAzol (Qiagen; Hilden, Germany) standard protocol. The RNA concentration of each sample was measured with the NanoDrop spectrophotometer. From total RNA, 1.5 μg was reverse transcribed using the miScript II RT Kit (Qiagen). The following Quantitect primers (Qiagen) were used: *Crh* (QT0029389) and *Crhr1* (QT00106232); and the following IDT primers were designed, verified for specificity and used for gene expression: *Nr3c1* (Forward): GGACCACCTCCCAAACTCTG; (Reverse): ATTGTGCTGTCCTTCCACTG; *Pgk* (Forward): TGCACGCTTCAAAAGCGCACG; (Reverse): AAGTCCACCCTCATCACGACCC. SYBR green PCR reactions were run in duplicate for all samples in the Rotor-Gene Real-Time PCR machine (Qiagen). Fold change relative expression was calculated using the ΔΔCt method, using the CT group as a reference and the *Pgk* as an endogenous control for analysis. Due to RNA degradation, two samples were excluded from the CT group. After the analysis of qPCR data, two outliers from the ELS group were excluded from the *Crh* gene expression.

### Corticosterone Assay

As a peripheral measure of HPA function, plasma corticosterone levels were measured. Following euthanasia by decapitation 30 min after the LD test, trunk blood was collected and centrifuged to obtain the plasma. A total of 5 μl of plasma was used for analyses in the Corticosterone Enzyme Immunoassay (Arbor Assays) ELISA kit, according to the manufacturer’s instructions. The optical density was analyzed at 450 nm wavelength in an ELISA plate reader, and the data was subsequently transformed into concentration (pg/ml) using standard curve parameters. Due to low-quality plasma samples, one animal from each group was excluded during the protocol.

### Statistical Analysis

SPSS software v.26 (SPSS, IL, USA) and GraphPad Prism 8 (GraphPad Software Inc., CA, USA) were used to perform statistical analysis. Differences between groups (CT × ELS) were analyzed by Student’s *t*-test. Shapiro–Wilk test of Normality was performed to verify the normality of data distribution. Pearson’s correlation analysis was performed to investigate associations between maternal care, behavioral, and biological data. To investigate the alterations in maternal care throughout the days and different time points, we performed a repeated-measures ANOVA. Tukey’s *post hoc* tests were conducted to identify the specific effects in a pairwise comparison. Data are presented as group mean ± standard error of the mean (SEM). Individuals are represented as dots in the graphs, and *p*-value <0.05 was considered as statistically significant.

## Results

### Body Weight

**Figure 2:**
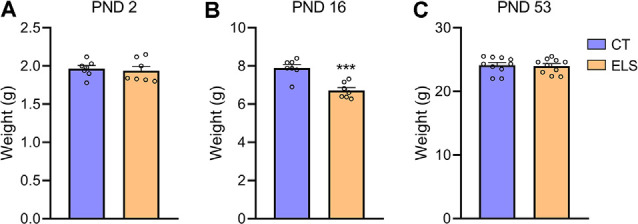
Body weight analysis; **(A)** PND 2; **(B)** PND 16; and **(C)** PND 53. Results are expressed as mean ± standard error of the mean (SEM); *n* = 7 litters per group (PND 2 and PND 16), and 10 animals on PND 53. ***p < 0.001 (Student’s *t*-test).

At PND 2 no significant weight difference was observed between groups (Figure 2A), but shortly after the end of the stress protocol (PND 16), animals exposed to ELS present a significant reduction in body weight when compared to CT animals (*t*_(12)_ = 4.933, *p* < 0.001; Figure 2B). Before the behavioral tasks (PND 53) no weight difference between groups was identified (Figure 2C).

### Maternal Behavior

**Figure 3:**
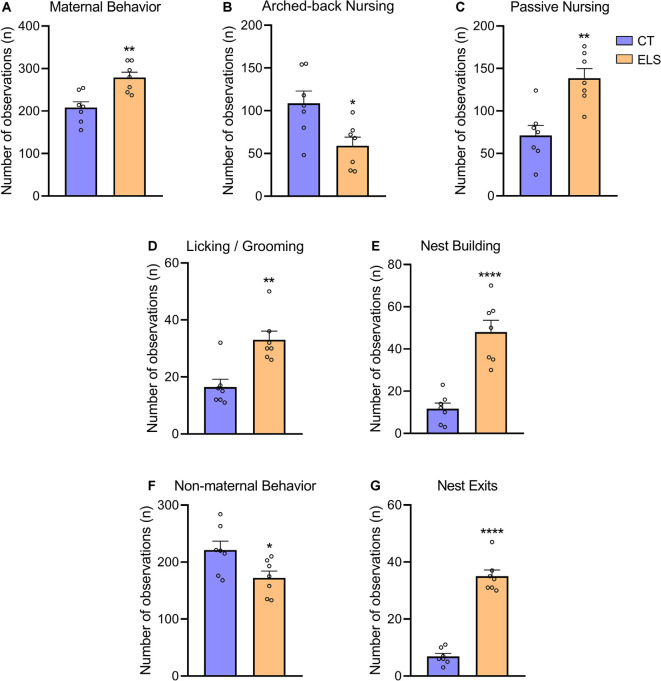
Maternal and non-maternal behavior analysis. **(A)** Total maternal behavior, which takes into account the sum of all maternal behaviors; **(B)** frequency of arched-back nursing throughout the days; **(C)** frequency of passive nursing throughout the days; **(D)** frequency of licking/grooming throughout the days; **(E)** frequency of nest building throughout the days; **(F)** total non-maternal behavior, which takes in account the sum of all non-maternal behaviors; and **(G)** frequency of exits from the nest throughout the days. Results are expressed as mean ± SEM; *n* = 7 litters per group. *p < 0.05; **p < 0.01; ****p < 0.0001 (Student’s *t*-test).

Regarding total maternal behavior, stressed dams showed significant increased frequency when compared to CT dams (*t*_(12)_ = 3.751, *p* = 0.002; Figure 3A). A repeated measures ANOVA indicated that independent of the day, this increase occurs when maternal behavior was analyzed in the morning (8 AM; *F*_(12)_ = 10.055, *p* = 0.008; Supplementary Figure 1A). However, *post hoc* analysis showed that at 1 PM this increase is only present at PND 5 (*p* = 0.016; interaction effect: *F*_(12)_ = 5.586, *p* = 0.036; Supplementary Figure 1A), and it is not persistent when maternal behavior was recorded during late afternoon (5 PM). When analyzing nursing behaviors, we observed that arched-back nursing, which is the most effective nursing posture was decreased in stressed dams (*t*_(12)_ = 2.800, *p* = 0.016; Figure 3B). A repeated measures ANOVA showed that this decrease in arched-back nursing occurred when maternal behavior was analyzed at 1 PM (*F*_(12)_ = 9.519, *p* = 0.009; Supplementary Figure 1B), but for 5 PM, this decrease only was present at PND 7 (*p* = 0.003) and PND 9 (*p* = 0.02; interaction effect: *F*_(12)_ = 10.203, *p* = 0.008; Supplementary Figure 1B). Moreover, passive nursing was increased in dams exposed to stress when compared to CT dams (*t*_(12)_ = 4.134, *p* = 0.001; Figure 3C). A repeated measures ANOVA indicated that this increase in the ELS group occurred at 8 AM (*F*_(12)_ = 5.183, *p* = 0.042; Supplementary Figure 1C), 1 PM (*F*_(12)_ = 10.696, *p* = 0.07; Supplementary Figure 1C), and 5 PM (*F*_(12)_ = 5.604, *p* = 0.036; Supplementary Figure 1C), independent of the day. Regarding Licking/grooming behavior, we observed a significant increase in stressed dams compared to CT dams (*t*_(12)_ = 4.014, *p* = 0.001; Figure 3D). A repeated measures ANOVA showed that independent of the day, this increase occurred both at 8 AM (*F*_(12)_ = 13.022, *p* = 0.004; Supplementary Figure 1D), and 1 PM (*F*_(12)_ = 12.003, *p* = 0.005; Supplementary Figure 1D), but it was not present at 5 PM Furthermore, we observed that stressed dams showed increased nest building frequency compared to CT dams (*t*_(12)_ = 5.887, *p* < 0.001; Figure 3E). A repeated measures ANOVA showed that at 8 AM, this increase was observed independent of the day (*F*_(12)_ = 4.88, *p* = 0.047; Supplementary Figure 1E). *Post hoc* analysis revealed an interaction effect for the other time points, which indicated that at 1 PM (*F*_(12)_ = 7.011, *p* = 0.021; Supplementary Figure 1E), this increase was present at PND 3 (*p* = 0.003), PND 5 (*p* = 0.029), PND 7 (*p* < 0.001), and PND 9 (*p* = 0.010). While at 5 PM (interaction effect: *F*_(12)_ = 7.959, *p* = 0.015; Supplementary Figure 1E), this effect was only present at PND 5 (*p* < 0.001) and PND 9 (*p* = 0.004).

In relation to total non-maternal behavior, stressed dams had a significant decrease compared to CT dams (*t*_(12)_ = 2.447, *p* = 0.03; Figure 3F). A repeated measures ANOVA followed by *post hoc* analysis, showed that this increase was only present when non-maternal behaviors were evaluated at 8 AM (interaction effect: *F*_(12)_ = 5.710, *p* = 0.034; Supplementary Figure 1F), during PND 5 (*p* = 0.01) and PND 7 (*p* = 0.013), but not effect was observed during any of the afternoon analysis. Moreover, we identified that stressed dams presented a fragmented maternal care pattern by increasing the number of exits from the nest (*t*_(12)_ = 11.47, *p* < 0.001; Figure 3G). A repeated measures ANOVA indicated that independent of the day, there was an increase in ELS non-maternal behavior at 1 PM (*F*_(12)_ = 45.929, *p* < 0.001; Supplementary Figure 1G). *Post hoc* analysis, revealed that this increase occurred when behaviors were evaluated at 8 AM (interaction effect: *F*_(12)_ = 11.584, *p* = 0.005; Supplementary Figure 1G), during PND 5 (*p* = 0.001), PND 7 (*p* = 0.005), and PND 9 (*p* = 0.001). Furthermore, similar effect was observed at 5 PM (interaction effect: *F*_(12)_ = 6.844, *p* = 0.023; Supplementary Figure 1G), but only during PND 3 (0.047) and PND 9 (*p* < 0.001).

All correlational analysis between maternal behavior, anxiety-like behavior *z*-score, and corticosterone levels can be found in Supplementary Table 1. We observed that arched-back nursing was negatively correlated with anxiety-like behavior *z*-score (*r* = −0.535; *p* = 0.015), and positively correlated with corticosterone levels (*r* = 0.680; *p* = 0.001). On the other hand, licking/grooming, nest building, total maternal behavior and exits from the nest displayed positive correlation with anxiety-like behavior *z*-score (*r* = 0.740, *p* < 0.001; *r* = 0.651, *p* = 0.001; *r* = 0.447, *p* = 0.047; *r* = 0.776, *p* < 0.001, respectively), and negative correlation with corticosterone levels (*r* = −0.593, *p* = 0.009; *r* = −0.624, *p* = 0.005; *r* = −0.481, *p* = 0.042; and *r* = −0.717, *p* < 0.001, respectively).

### Anxiety-Like Behavior

**Figure 4:**
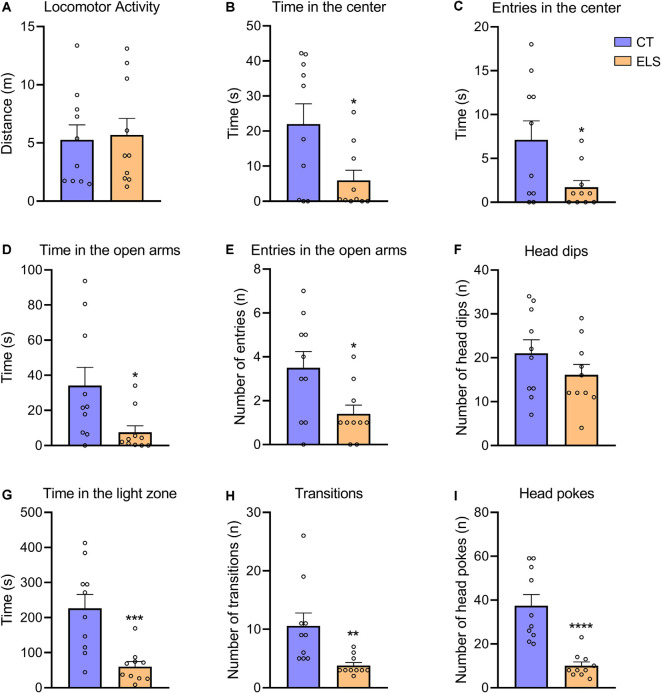
The measure of anxiety-like behavior in the Open Field (OF), Elevated Plus Maze (EPM), and light/dark test (LD). **(A)** Distance exploring the OF apparatus; **(B)** time spent in the center of the OF apparatus; **(C)** number of entries in the center of the OF apparatus; **(D)** time spent in the open arms of the EPM apparatus; **(E)** number of entries in the open arms of the EPM apparatus; **(F)** number of head dips (risk assessment) in the open arms of the EPM apparatus; **(G)** time spent in the light zone of the LD apparatus; **(H)** number of transitions between zones of the LD apparatus; and **(I)** number of head pokes (risk assessment) between zones of the LD apparatus. Results are expressed as mean ± SEM; *n* = 10 per group. *p < 0.05; **p < 0.01; ***p < 0.001; ****p < 0.0001 (Student’s *t*-test).

Regarding the OF test, there was no significant difference between groups in locomotor activity (Figure 4A). However, animals exposed to ELS showed decreased time spent in the center and less entries in the center of the apparatus compared to CT animals (*t*_(18)_ = 2.484, *p* = 0.023; Figure 4B; *t*_(18)_ = 2.346, *p* = 0.03; Figure 4C, respectively).

In the EPM test, we identified a significant decrease in time spent in the open arms and number of entries in the open arms in ELS animals (*t*_(18)_ = 2.409, *p* = 0.026; Figure 4D; *t*_(18)_ = 2.512, *p* = 0.021; Figure 4E, respectively). The number of head dips in the open arms did not present statistically significant differences between groups (Figure 4F).

In relation to the LD test, ELS animals presented a significant decrease in time spent in the light zone, number of transitions and total number of head pokes between zones compared to CT animals (*t*_(18)_ = 3.949, *p* < 0.001; Figure 4G; *t*_(18)_ = 3.034, *p* = 0.007; Figure 4H; *t*_(18)_ = 5.059, *p* < 0.001; Figure 4I, respectively).

**Figure 5:**
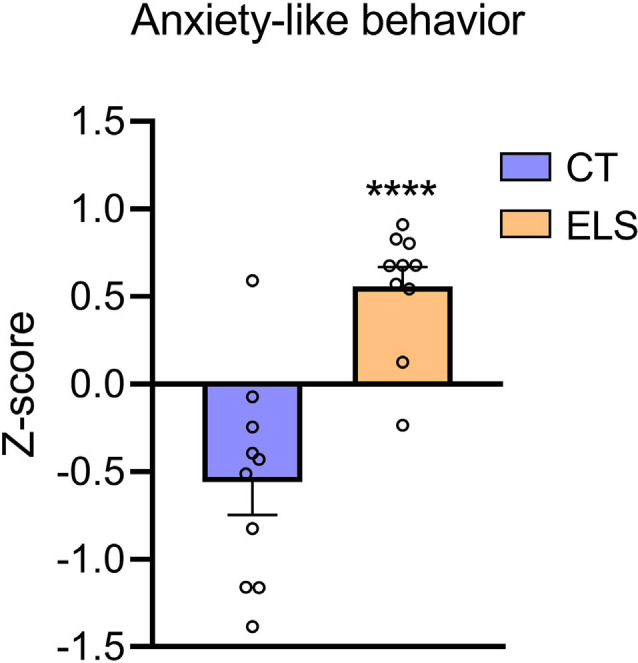
Anxiety-like behavior *z*-score. *Z*-score is calculated using the time spent in the center of the OF, time spent in the open arms of the EPM, and time spent in the bright chamber of the LD. Results are expressed as mean ± SEM; *n* = 10 per group. ****p < 0.0001 (Student’s *t*-test).

Regarding the *z*-score, which took in account results from all three tests combined, ELS animals showed an increased score compared to CT group (*t*_(18)_ = 5.111, *p* < 0.001; Figure 5).

### Gene Expression Analysis

**Figure 6:**
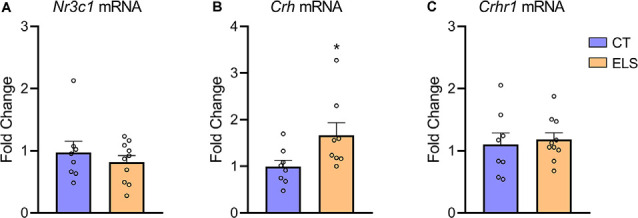
Hypothalamic gene expression of hypothalamic-pituitary-adrenal (HPA) markers. **(A)** *Nr3c1* gene expression; **(B)** *Crh* gene expression; and **(C)** *Crhr1* gene expression. Results are expressed as mean ± SEM; *n* = 8–10 per group. *p < 0.05 (Student’s *t*-test).

When assessing *Crh* mRNA expression in the hypothalamus, we identified an increase in ELS animals compared to CT animals (*t*_(14)_ = 2.211, *p* = 0.044; Figure 6B). However, there was no significant difference in *NR3C1* and *Crhr1* mRNA expression between groups (Figures 6A,C, respectively).

### Corticosterone Analysis

**Figure 7:**
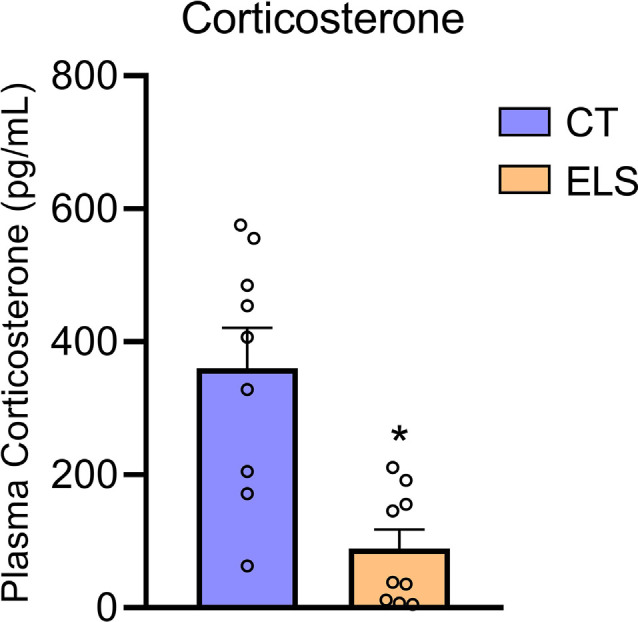
Peripheral HPA response analysis. Plasmatic corticosterone levels. Results are expressed as mean ± SEM; n 6–9 per group. *p < 0.05 (Student’s *t*-test).

Regarding plasma levels of corticosterone, we identified a significant decrease in animals exposed to ELS protocol compared to the CT group (*t*_(16)_ = 4.082, *p* < 0.001; Figure 7). Furthermore, we observed a negative correlation between anxiety-like behavior *z*-score data and plasmatic corticosterone levels (*r* = −0.571, *p* = 0.013; Table 1).

**Table 1:** Pearson’s correlation index between anxiety-like behavior *z*-score, *Nr3c1*, *Crh*, *Crh*r1, and Corticosterone.

	Anxiety-like behavior *z*-score		*Nr3c1*		*Crh*		*Crhr1*		Corticosterone	
	*r*	*p*	*r*	*p*	*r*	*p*	*r*	*p*	*r*	*p*
Anxiety-like behavior *z*-score
*Nr3c1*	0.025	0.919								
*Crh*	0.095	0.705	−0.334	0.206						
*Crhr1*	−0.332	0.177	−0.038	0.880	−0.304	0.251				
*Corticosterone*	−0.571*	0.013*	−0.017	0.945	−0.361	0.168	−0.035	−0.893		

Note: *p < 0.05.

## Discussion

This study aimed to investigate the effects of LB and MS models combined on classical anxiety-like behavior tasks and hypothalamic gene expression of targets related to HPA axis functioning (*Crh*, *Crhr1*, and *Nr3c1*). Overall, our findings suggested a significant anxious effect of ELS on the main anxiety-like parameters analyzed in the OF (time and entries in the center), EPM (time and entries in the open arms), and LD (time in the light zone, transitions and head pokes). The anxiety phenotype evaluated through the composite *z*-score analysis revealed a higher anxious response of ELS animals in comparison to the CT group. Results from mRNA gene expression revealed significant changes in *Crh* mRNA levels, with increased gene expression in the hypothalamus, while the measure of plasma CORT levels indicated a decrease in ELS animals. Also, our study assessed maternal behavior of dams during early development, in which we observed significant changes in both quantitative and qualitative patterns of maternal care among ELS dams. Especially, we observed an unbalance between arched-back and passive nursing (e.g., decrease of arched-back nursing and increase of passive nursing), despite the increase of licking/grooming, nest building, and total maternal behavior in ELS dams. Moreover, ELS dams presented an increase in the number of exits from the nest, which is considered a fragmented maternal care pattern.

Here, we adopted a new approach to induce a translational and reliable ELS effect by combining MS and LB. The insights for combining ELS protocols derived from discussions expanded by McLaughlin et al. (2014) that proposed a two-dimensional Threat/Deprivation system in quantification and qualification of early stress exposure. Threatening adversities could trigger fear and physical stress, in both relational and environmental contexts. On the other side, deprivation is related to an insufficient early stimulation, which includes lack of maternal support or appropriate resources and condition for adequate development (i.e., reproducing poverty conditions). In this sense, the combination of both LB and MS could have complementary roles, since LB is suggested to induce significant levels of deprivation and low levels of threat, while MS is associated with intense threat during separation periods (White and Kaffman, 2019). It has already been demonstrated that LB leads to decreased quality of maternal care, which reproduces a lack of early stimulus (Rice et al., 2008). However, during the MS paradigm, the threat tends to raise in response to the novel environment. On the other hand, in a recent review from our laboratory (Orso et al., 2019), we found that MS alone induces an increase in the levels of licking/grooming post-reunion. This counterbalance behavior (increase in maternal care) could attenuate the deprivation characteristics of the MS model, and possibly minimize the effects of the separation period (Davis et al., 2020; Wang et al., 2020). Furthermore, during MS, pups are also exposed to an unknown environment, which is full of novel cues that can also have a direct impact on neurodevelopment. The combination of several factors, such as threat/fear, novel cues, and possible compensatory maternal care displays the challenges in investigating and replicating ELS data in rodents.

Due to the major importance of maternal care for offspring neurodevelopment, we investigated maternal behavior during the ELS protocol. Our analyses suggested significant changes in the pattern of maternal care. Overall, ELS animals showed an increase in the frequency of total maternal behavior, while total non-maternal behavior was decreased. The idea of a compensatory mechanism *via* maternal care alterations can be supported by our data indicating that total maternal care was increased immediately after MS, but it is not persistent during 5 PM analysis. Moreover, an increase in licking/grooming was identified, which corroborates with the compensation hypothesis previously proposed (Orso et al., 2018). These alterations were also followed by an increased number of exits from the nest in animals exposed to ELS, which replicates results usually found in response to LB exposure (Ivy et al., 2008; Walker et al., 2017). Moreover, ELS dams displayed increased nest-building frequency, which indicates an excessive effort to increase nest quality during stressful conditions. We observed that this increase occurred following MS protocol, which reinforces the importance of an adequate nest for pup neurodevelopment. Moreover, ELS dams presented a reduction in the frequency of arched-back nursing, which significantly decreases the quality of maternal care, since this behavior plays a major role in offspring development (Champagne et al., 2003, 2007). Interestingly, this reduced frequency does not only occur immediately after MS (1 PM) but also persists during late afternoon (5 PM) analysis. Our correlational analysis supported this hypothesis, which showed that animals exposed to low levels of arched-back nursing displayed a long-lasting increase in anxiety-like behavior and blunted corticosterone response during early adulthood. Furthermore, we showed that other maternal behaviors, such as nest building and licking/grooming were positively correlated with increased anxiety-like behavior, which could be associated with an attempt of the dam to reduce the deleterious effects of ELS. Thus, using the combination of stressors, we might induce a fragmented and unpredictable maternal care pattern, reproducing a chronic stress condition in both dams and offspring (Walker et al., 2017).

Regarding the behavioral phenotype evaluation, we observed a robust increase in anxiety-like behavior in animals exposed to ELS. The anxiogenic response was identified in all three tasks (OF, EPM, and LD). Moreover, our composite *z*-score analysis sustained the anxiety phenotype as a markable trait in ELS animals. Together these findings corroborate with a recent study, which showed that LB and MS combined resulted in a robust increase in anxiety-like behavior, but LB alone was not sufficient to generate an anxiogenic phenotype (Johnson et al., 2018). Furthermore, several studies have also previously shown that animals reared in LB conditions did not present an increase in anxiety-like behavior during adulthood (Brunson et al., 2005; Rice et al., 2008; Goodwill et al., 2019; Davis et al., 2020; Wang et al., 2012). Those results are not restricted to LB protocol, since they are supported by a range of studies suggesting inconclusive effects of MS. Even though the evidence is indicating that MS exposure may increase anxiety-like behavior (Wei et al., 2010; Malcon et al., 2020), several studies do not replicate those traditional long-term anxiogenic effects following MS (Hulshof et al., 2011; Tan et al., 2017; He et al., 2020). Recently, a systematic review and meta-analytic study reported that there is extensive variability among studies that investigated the impact of MS on OF and EPM tests, which indicates that a better protocol standardization is necessary (Wang et al., 2020).

Animals exposed to ELS showed a blunted corticosterone response 30 min following the LD test. While evidence indicates that ELS exposure can increase corticosterone levels (Rice et al., 2008; Viola et al., 2019), our results may be explained by the high degree of stress applied to the offspring and their dams. This data is supported by a hypothesis which argues that blunted cortisol levels may occur as an adaptation of the sustained periods of HPA axis hyper-reactivity (Trickett et al., 2010; Bunea et al., 2017). Moreover, a recent study with humans reported that ELS severity was negatively correlated with long-term cortisol levels, which shows that a severely stressful event early in development could indeed lead to a blunted cortisol response during adulthood (Zhang et al., 2019). Here, we also showed that plasmatic corticosterone levels were negatively correlated with the anxiety-like behavior *z*-score, which indicates that blunted corticosterone levels were associated with increased anxiety like-behavior. This result could indicate that maintaining adequate corticosterone levels is required to cope with the challenges generated by the behavioral tasks (Cohen et al., 2006). Moreover, previous data have shown that blunted corticosterone levels were associated with HPA axis malfunctioning, which is known to be critical during fight-or-flight situations (Algamal et al., 2018). Furthermore, we also observed an increase in the hypothalamic *Crh* mRNA levels of ELS animals. Following our data, a previous study reported that HPA axis overactivation through ELS exposure may lead to a long-term increase in CRH levels in both the hypothalamus and amygdala (Lai and Huang, 2011). Furthermore, it has been shown that CRH overproduction in transgenic unstressed mice resulted in increased anxiety-like behavior (Stenzel-Poore et al., 1994; van Gaalen et al., 2002). Furthermore, naive CRHR1 knockout animals have been associated with reduced anxiety-like behavior (Smith et al., 1998).

Certain limitations should be considered when interpreting the current work. First, due to the novelty of this ELS model, we opted to perform this study only with male animals. We acknowledge the importance of investigating ELS sex-related differences, so once this combined model of MS and LB is better understood, future studies should seek to investigate neurobiological and behavioral sex differences. Second, even though our study showed a robust effect of the combined ELS model on anxiety-like behavior, it would be important to investigate a direct comparison between MS and LB alone and the combined model of ELS on anxiety-like behavior. For that reason, a future study that will investigate the differences in anxiety-like behavior provoked by these three models is planned. Finally, our study was performed with a single cohort of animals, in which all three behavioral tests and biomolecular analyses were performed. So, we cannot conclude how ELS independently affected gene expression and plasma corticosterone levels without potential responsiveness effect from the behavioral tasks. This strategy was used to reduce the number of animals utilized (Jonasson, 2005), as indicated by the 3Rs (Replacement, Reduction, and Refinement) principle (Flecknell, 2002). The animals were also exposed from less to most stressful tests to have minimal impact on the behavioral results.

In conclusion, exposure to a combined model of MS and LB provoked a long-term increase in anxiety-like behavior in the OF, EPM, and LD tasks. This combined model also resulted in a fragmented and low-quality maternal care, due to an increase in the number of exits from the nest and decreased arched-back nursing frequency. Moreover, a robust disruption in the HPA axis function was identified. Animals exposed to ELS showed a long-term increase in hypothalamic *Crh* mRNA levels and a blunted plasmatic corticosterone response. We believe that the proposed combined model of ELS may generate a translational and reliable approach to mimic the characteristics of human early life adverse conditions, and increase the replicability of ELS preclinical studies.

## Data Availability Statement

The raw data supporting the conclusions of this article is available in the Supplementary Material.

## Ethics Statement

The animal study was reviewed and approved by Committee of Ethics on the Use of Animals (CEUA) of PUCRS.

## Author Contributions

RO: conceptualization, methodology, investigation, formal analysis, and writing—original draft. KC: conceptualization, investigation, and writing—original draft. EK-F: investigation. LW-S: conceptualization and methodology. ST: writing—original draft and writing—review and editing. RG-O: writing—review and editing, supervision, project administration, and funding acquisition. All authors contributed to the article and approved the submitted version.

## Conflict of Interest

The authors declare that the research was conducted in the absence of any commercial or financial relationships that could be construed as a potential conflict of interest.
