# Supplementary material for: Maternal Separation Combined With Limited Bedding Increases Anxiety-Like Behavior and Alters Hypothalamic-Pituitary-Adrenal Axis Function of Male BALB/cJ Mice
Source: Front Behav Neurosci. 2020 Nov 12;14:600766. doi: 10.3389/fnbeh.2020.600766 (PMC7693708; doi:10.3389/fnbeh.2020.600766)
Supplement: Supplementary file 1 [file Table_1.docx]

**Table S1.** Pearson’s correlation index between maternal behavior, anxiety-like behavior Z-score and Corticosterone.

|  | **Arched-back nursing** | | **Passive nursing** | | **Licking/**  **Grooming** | | **Nest building** | | **Maternal behavior** | | **Nest exits** | | **Non-maternal behavior** | |
| --- | --- | --- | --- | --- | --- | --- | --- | --- | --- | --- | --- | --- | --- | --- |
|  | *r* | *p* | *r* | *p* | *r* | *p* | *r* | *p* | *r* | *p* | *r* | *p* | *r* | *p* |
| **Anxiety-like behavior**  **Z-score** | **- 0.535** | **0.015*** | 0.269 | 0.250 | **0.740** | **<0.001*** | **0.651** | **0.001*** | **0.447** | **0.047*** | **0.776** | **<0.001*** | - 0.244 | 0.298 |
| **Corticosterone** | **0.680** | **0.001*** | - 0.428 | 0.076 | **- 0.593** | **0.009*** | **- 0.624** | **0.005*** | **- 0.481** | **0.042*** | **- 0.717** | **<0.001*** | 0.316 | 0.200 |


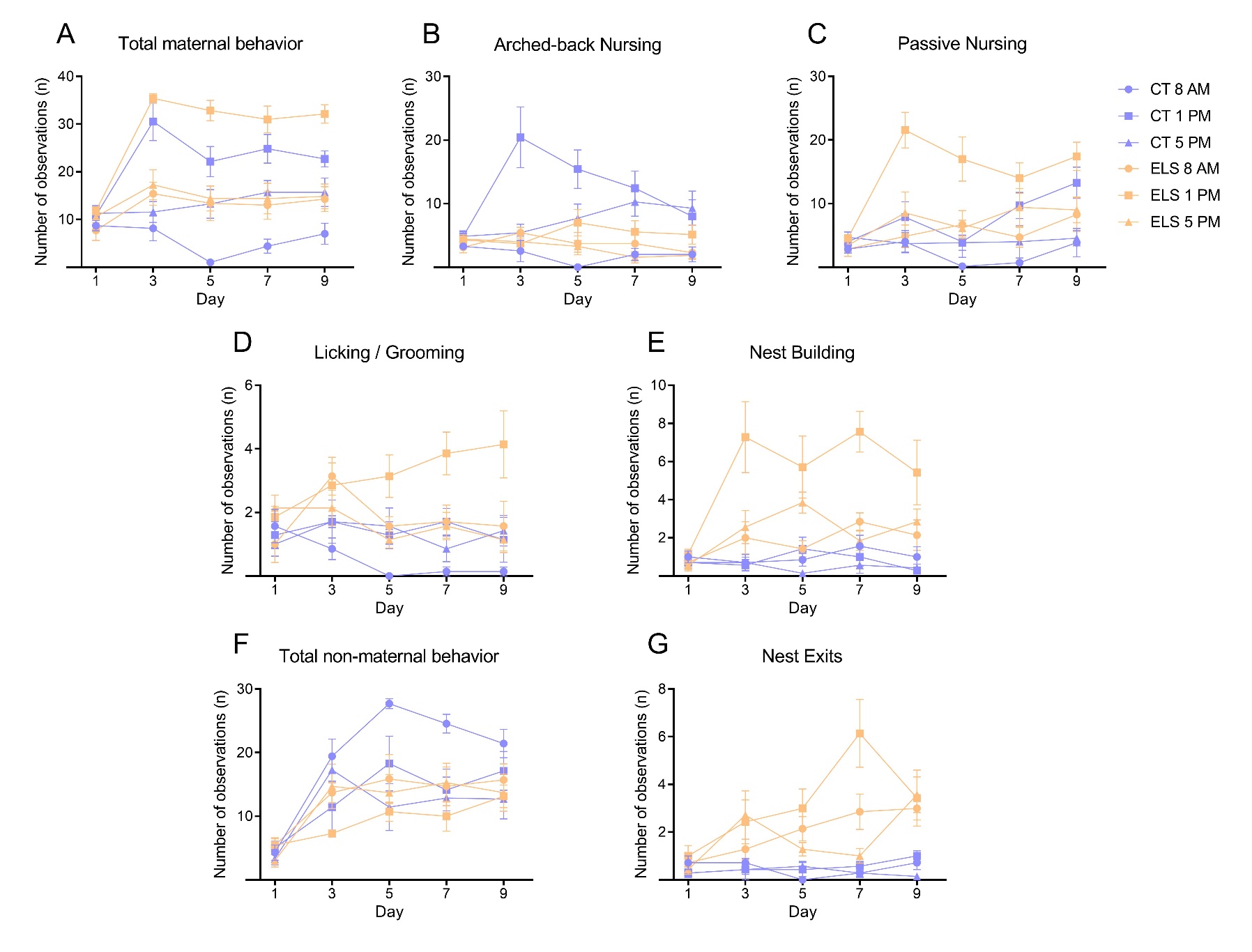


**Figure S1**. Analysis of maternal and non-maternal behaviors throughout each day and specific time points. A) Total maternal behavior during PND 1, PND 3, PND 5, PND 7, and PND 9 at 8 a.m., 1 p.m., and 5 p.m.; B) Arched-back nursing during PND 1, PND 3, PND 5, PND 7, and PND 9 at 8 a.m., 1 p.m., and 5 p.m.; C) Passive nursing during PND 1, PND 3, PND 5, PND 7, and PND 9 at 8 a.m., 1 p.m., and 5 p.m.; D) Licking/grooming during PND 1, PND 3, PND 5, PND 7, and PND 9 at 8 a.m., 1 p.m., and 5 p.m.; E) Nest building during PND 1, PND 3, PND 5, PND 7, and PND 9 at 8 a.m., 1 p.m., and 5 p.m.; F) Total non-maternal behavior during PND 1, PND 3, PND 5, PND 7, and PND 9 at 8 a.m., 1 p.m., and 5 p.m.; G) Exits from the nest during PND 1, PND 3, PND 5, PND 7, and PND 9 at 8 a.m., 1 p.m., and 5 p.m.. Results are expressed as mean ± SEM; n = 7 litters per group. (Repeated Measures ANOVA followed by Tukey’s post-hoc when necessary).
